# Acquired hemophilia A (AHA) due to anti‐SARS‐CoV‐2 vaccination: A systematic review

**DOI:** 10.1002/jha2.604

**Published:** 2023-03-10

**Authors:** Fnu Amisha, Prachi Saluja, Paras Malik, Frits Van Rhee

**Affiliations:** ^1^ Department of Internal Medicine University of Arkansas for Medical Sciences Little Rock Arkansas USA; ^2^ Department of Internal Medicine University of Arkansas for Medical Sciences Little Rock Arkansas USA; ^3^ Department of Internal Medicine Jacobi Medical Center‐Albert Einstein College of Medicine Bronx New York USA; ^4^ Division of Hematology/Oncology Department of Internal Medicine University of Arkansas for Medical Sciences Little Rock Arkansas USA

**Keywords:** acquired hemophilia A, AHA, BNT162b2 vaccine, COVID‐19 vaccine, mRNA‐1273 vaccine

## Abstract

Vaccination against SARS‐CoV2 has been the largest vaccination campaign over the past two decades. The aim of this study is to qualitatively assess the reported cases of acquired hemophilia A (AHA) that developed after COVID‐19 vaccination to further elaborate on incidence, presentation, treatment, and outcomes.We queried Medline (PubMed), Google Scholar, and Embase databases to find reported cases of AHA after COVID‐19 vaccines. We found 14 studies (19 cases) for this descriptive analysis. Most patients were elderly (mean age 73 years) and males (*n* = 12) with multiple comorbidities. All cases developed after mRNA vaccines ‐ BNT162b2 Pfizer‐BioNTech (*n* = 13) and mRNA‐1273 Moderna (*n* = 6). All except one patient were treated, with the most common therapy being a combination of steroids, immunosuppression, and rFVIII (*n* = 13). Two patients died due to acute respiratory distress, and gall bladder rupture with persistent bleeding, respectively. While evaluating a patient with bleeding diathesis after COVID‐19 vaccination, AHA should be kept in the differential diagnosis. Given the low incidence, we believe that the benefit of vaccination still outweighs the risk of disease acquisition.

1

Acquired hemophilia A (AHA) is a rare autoimmune condition caused by the formation of neutralizing autoantibodies against coagulation factor VIII. Annual incidence of AHA is approximately 1.5 cases per million people [1]. Majority (approximately 51.9%) of AHA cases are idiopathic [2]. However, AHA has been associated with other autoimmune conditions, medications, lymphoproliferative disorders, infections, graft‐versus host disease, surgery, or the postpartum period. Occasionally the onset has also been linked with infections and vaccines (seasonal flu, HINI) [3, 4]. SARS‐CoV2‐2 infection leads to proinflammatory or prothrombotic state, but rare instances of hemorrhagic complications including de novo AHA have been reported as well. Vaccination against SARS‐CoV2 has been the largest vaccination campaign over the past two decades. There are two types of anti‐SARS‐CoV2‐2 vaccines that have been approved to curb the ongoing pandemic. The nucleoside‐modified messenger RNA (BNT162b2 Pfizer‐BioNTech and mRNA‐1273 Moderna) and adenovirus‐based DNA vector (ChAdOx1‐S AstraZeneca and Ad26.COV2‐S Johnson & Johnson's Janssen) vaccines both encode the same SARS‐CoV‐2 spike glycoprotein S1, which has been linked to new or recrudescent immune hematologic complications with the natural infection. Since the emergence of anti‐SARS‐CoV‐2 vaccines, majority of the vaccine‐related adverse events that are reported were minor but occasionally they have been related to immune‐mediated hematologic phenomena such as Immune thrombocytopenic purpura, thrombotic microangiopathies, Paroxysmal nocturnal hemoglobinuria, vaccine‐induced thrombotic thrombocytopenia (VITT), aplastic anemia etc. [5, 6, 7]. Hence, postmarketing surveillance to report rare vaccine‐associated adverse events is very essential. The aim of this study is to qualitatively assess the reported cases of AHA that developed after COVID‐19 vaccination to further elaborate on incidence, presentation, treatment, and outcomes

For this systematic review, we did an electronic search in PubMed, Google Scholar, and Embase databases to find reported cases of AHA after COVID‐19 vaccine using the keywords (either Mesh or Supplementary concept) ‐ “COVID‐19 vaccines” or “COVID‐19 vaccine booster shot” or “2019‐nCoV vaccine mRNA‐1273” or “ChAdOx1 nCoV‐19” or “BNT162 vaccine” OR “Ad26COVS1” and “Factor VIII deficiency, acquired” OR “hemophilia A, acquired” OR “hemophilia A” from inception till April, 2022. The study was performed following the preferred reporting items for systematic reviews and meta‐analyses statement guidelines [Figure 1] [8]. The title and abstracts were independently screened by two authors (FA, PM). After removing duplicated articles, data were extracted from the eligible studies b two independent authors (FA, PS). Any discrepancy was resolved with the consensus of authors. Additional articles were included by cross‐referencing the reference list of the included articles. The inclusion criteria were as follows: (1) Case reports, case series, cohort studies, commentaries, abstracts to conference, or letter to editor. (2) Demographic data of individual patients were reported. (3) Laboratory data of individual patients were reported. Articles not reporting individual patient data and non‐English publications were excluded from this review. In the end, 14 studies (19 cases) were included in this study. The methodological quality of the case reports and case series included in the study were assessed using the tool proposed by Murad et al. based on four domains—selection, ascertainment, causality, and reporting [9]. The mean assessment score was 4.9. The data collected from each manuscript are summarized in (Table 1). The variables collected include (1) author name and country of origin, (2) age and sex of patient, (3) comorbid conditions, (4) vaccine type and dose, (5) time to first symptom onset and presentation, (6) laboratory findings, and (7) treatment and outcomes. Quantitative and qualitative analysis was done for synthesis of results.

**FIGURE 1:**
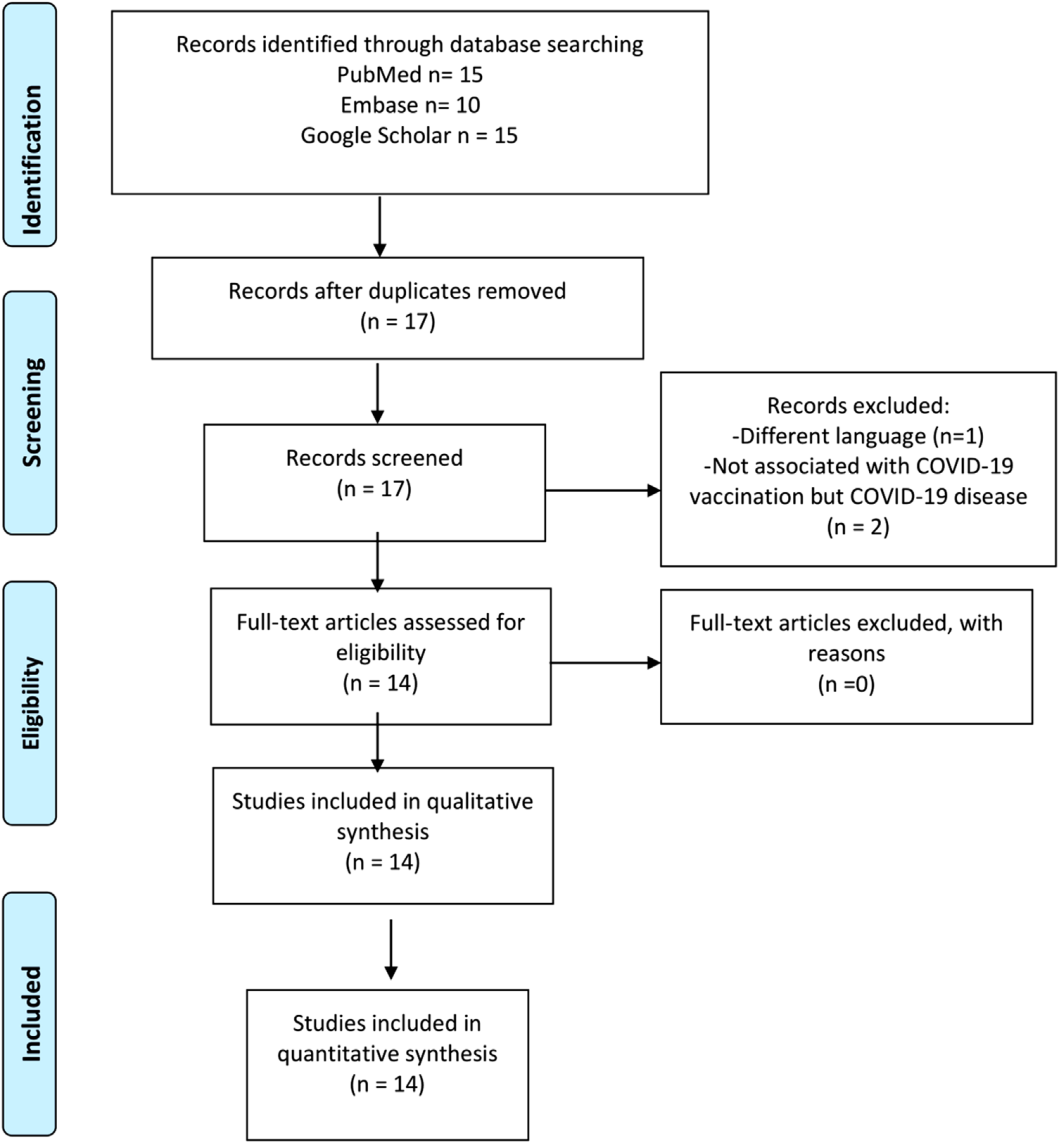
Preferred reporting items for systematic reviews and meta‐analyses statement (PRISMA) flow chart for this study

**TABLE 1:** Summarizing the data extracted from individual study for pooled patient analysis

Reference Number	Study	Qualitative assessment of the case report S‐Selection A‐Ascertainment C‐Causality R‐Reporting (Total score = 8)	Place	Patient age, sex	Comorbid conditions	Vaccine	Vaccine dose	Presentation	Time from last administered vaccine dose to symptom onset	aPTT (sec)	FVIII inhibitor titers (Bu/ml)	Factor VIII activity (%) or titer (IU/ml) as reported	Treatment	Outcome
1.	Al Hennawi et al. [2]	S1A2C0R1 = 4	Saudi Arabia	75, M	T2DM, HTN, HPLD, BPH, CAD	Pfizer	2	Spontaneous Ecchymosis with R forearm and flank hematomas	90 days	89.2	318	<1%	rFVIII followed by prednisone 80 mg for 3 days, rituximab 375 mg/m^2^, cyclophosphamide 750 mg/m^2^, cyclosporine 25 mg twice daily	NA
2.	Farley et al. [10]	S1A2C1R1 = 5	United States	67, M	HTN, pulmonary sarcoidosis	Pfizer	2	Spontaneous left thigh hematoma	19 days	72	110	<1%	FEIBA at 4500 u/kg every 8 h, oral prednisone (1 mg/kg), rituximab (375 mg/m^2^ × four doses) and recombinant FVIIa	FVIII inhibitor decreased to 8 Bu/ml after 2 weeks and FVIII was 171% after 34 days
3.	Cittone et al. [11]	S1A2C1R1 = 5	Switzerland	72, F	Multiple comorbidities including arterial disease	Moderna	1	Spontaneous cutaneous hematomas	14 days	184	12.4	Not detectable	rFVIII × 7 days with tranexamic acid with prednisone (100 mg/day) with rituximab 375 mg/m^2^ weekly (four doses)	FVIII activity increased to 5%, whereas FVIII inhibitor decreased to 5.6 Bu/ml after 3 weeks
4.	Cittone et al. [11]	S1A2C1R1 = 5	Switzerland	86, F	Third degree AV block with pacemaker and moderate to severe aortic stenosis	Moderna	2	Right hemothorax after rib fractures following a mechanical fall	21 days	Prolonged	1.01	FVIII: C 23%	rFVIII, which was switched to APCC to control local bleeding, prednisone (1 mg/kg)	FVIII activity increased to 178% after 17 days
5.	Cittone et al. [11]	S1A2C1R1 = 5	Switzerland	85, M	HTN, CAD, PAD, renal and carotid stenosis	Moderna	1	Spontaneous hematomas of right thigh, iliopsoas, and forearm with right knee hemarthrosis	7 days	49	2.2	Not detectable	rFVIII then APCC with prednisone (100 mg/day) with Rituximab	After 3.5 weeks, acute gall. Bladder rupture with active bleeding, arterial embolization was done but patient refused any surgery with subsequent death
6.	Portugese et al. [12]	S1A2C1R1 = 5	United States	76, F	Asthma, Raynaud's phenomenon, hiatal hernia, Von Willebrand disease	Moderna	2	Ecchymosis in upper extremities, melena, and syncope	4 days	122	11.2	<3%	vWF/FVIII replacement therapy with Humane‐P 2290 U every 12 h × 4 doses along with IVIg and methyprednisone	Elevation in FVIII and reduction of inhibitor levels <0.5 Bu/ml after 24 days
7.	Radwi and Farsi [13]	S1A2C1R1 = 5	Saudi Arabia	69, M	T2DM, HTN, prostate adenocarcinoma in remission	Pfizer	2	Spontaneous bruising of left wrist after first dose and then multiple on arms and legs after second dose with minor trauma‐induced rectus femoris hematoma	9 days	115.2	80	1%	Prednisone (1 mg/kg ) for 4 weeks	FVIII increased to 5% and inhibitor decreased to 2 after 4 weeks
8.	Ai Vuen L et al. [14]	S1A2C1R1 = 5	Malaysia	80, M	T2DM, HTN, HPLD, CKD Stage3a, BPH, glaucoma	Pfizer	1	Bruising upper and lower extremities for 4 days	14 days	78.7	7.5	6.7%	Tranexamic acid with single dose recombinant FVIII who methylprednisolone 500 mg/day × 3 days followed by taper and azathioprine 100 mg daily	FVIII levels were normal, and inhibitors were undetectable after 6 weeks
9.	Lemoine et al. [15]	S1A2C1R1 = 5	United States	70, M	Polymyalgia rheumatic and Hepatitis C	Moderna	1	Right extremity bruising	8 days	57.5	39.9	0.03 IU/ml	FEIBA 5000 IU every 6 h alternating every 3 h with recombinant FVIII 90 mcg/kg every 6 h for the first 24 h then FEIBA alone with prednisone 1 mg/kg and cyclophosphamide 2 mg/kg daily.	FVIII activity increased to 0.07 IU/ml in 6 days and inhibitor decreased to 11.4 BU
10.	Gonzalez et al. [16]	S1A2C0R1 = 4	NA	43, F	None	Pfizer	2	Dark purple lesions in bilateral extremities for 1 month	21 days	86.10	78.4	<5%	IV methylprednisolone and Rituximab	NA
11.	Soliman DS et al. [17]	S1A2C1R1 = 5	Qatar	39, F	None	Pfizer	1	Lower abdominal pain and frank hematuria for 3 days	10 days	72.2	17.2	2%	None	FVIII recovered 78.8% 2 months after vaccine spontaneously
12.	Leone et al. [18]	S1A2C1R1 = 5	Italy	86, M	Polymyalgia rheumatica (remission)	Pfizer	2	Spontaneous disseminated hematomas with severe anemia	14 days	aPTT ratio (reference interval 0.8–1.2) 1.91	2.1	FVIII:C (reference interval 0.5–1.5 IU/ml) 0.06 IU/ml	Methylprednisolone 1 mg/kg/day	FVIII:C 0.82 IU/ml and undetectable inhibitor at 7 months
13.	Leone et al. [18]	S1A2C1R1 = 5	Italy	73, F	Rheumatoid arthritis, Sjogren syndrome (remission)	Pfizer	2	Tongue hematoma after first dose and jaw and knee hematomas after second dose	14 days	aPTT ratio (reference interval 0.8–1.2) 2.1	0.8	0.05 IU/ml	Methylprednisolone 1 mg/kg/day	FVIII:C 0.1.62 IU/ml and undetectable inhibitor at 5 months
14.	Leone et al. [18]	S1A2C1R1 = 5	Italy	67, M	None	Pfizer	2	Hematoma of tongue extending in the cervical region	49 days	aPTT ratio (reference interval 0.8–1.2) 2.55	2.5	0.06 IU/ml	Prednisone (I mg/kg/day), recombinant FVIII (90 mg/kg every 6 h) and cyclophosphamide (1 mg/kg/day )	FVIII:C 1.21 IU/ml and undetectable inhibitor at 6 days
15.	Leone et al. [18]	S1A2C1R1 = 5	Italy	77, M	Bladder cancer	Pfizer	2	Hematuria	52 days	aPTT ratio (reference interval 0.8–1.2) 3.61	6.9	0.02 IU/ml	Methylprednisolone (1 mg/kg/day), recombinant FVIII ( 90 mg/kg every 6 h) and rituximab	FVIII:C 0.96 IU/ml and undetectable inhibitor but died of severe sepsis and respiratory complications
16.	Murali et al. [19]	S1A2C1R1 = 5	Australia	95, F	Dementia, HTN, CHF, depression, breast cancer in remission	Pfizer	1	Bruising in extremities 1 week after first dose and then spontaneous persistent bleeding from dorsum of right hand 3 weeks after second dose	7 days	83	5.4	< 0.01 IU/ml	IV rituximab 100 mg weekly × 4 weeks	FVIII levels became normal 0.68 IU/ml in 22 days
17.	Fu PA et al. [20]	S1A2C1R1 = 5	Taiwan	77, M	NA	Moderna	2	Multiple ecchymosis of b/l arms and legs with hemorrhagic blisters	21 days	97.3	71.6	0.6%	FEIBA (90 mcg/kg) × two doses, prednisolone 1 mg/kg/day and cyclophosphamide 100 mg/day	FVIII activity increased to 9% and inhibitor decreased to 49 BU after 4 weeks
18.	Plüß M et al. [21]	S1A2C1R1 = 5	Germany	72, M	BPH, carpal tunnel syndrome	Pfizer	3	Bruises on arms, legs and trunk with forehead nodule that was pleomorphic dermal sarcoma	9 days	164.6	158.6	<0.1%	rFVIII, prednisone 100 mg/day, rituximab weekly (375 mg/m^2^) and oral cyclophosphamide 150 mg daily	FVIII activity 8% and inhibitor 20.6 BU after 3 weeks
19.	O'shea et al. [22]	S1A2C1R1 = 5	Ireland	72, M	T2DM, HTN, Prostate carcinoma	Pfizer	1	Arm and thigh bruising	7 days	71	70	0.01 IU/ml	rFVIII 4500 units x 1 dose, steroid, and rituximab weekly 375 mg/m^2^	FVIII was normal and inhibitor was negative after 6 weeks

Abbreviations: APCC, activated prothrombin complex concentrate; ARDS, acute respiratory distress syndrome; BPH, benign prostate hyperplasia; BU, Bethesda units; CAD, coronary artery disease; CHF, congestive heart failure; CKD, chronic kidney disease; FEIBA, factor eight inhibitor bypass activity; HPLD, hyperlipidemia; HTN, hypertension; PAD, peripheral arterial disease; rFVIII, recombinant Factor VIII; vWF, Von Willebrand factor; T2DM, type 2 diabetes mellitus.

The median age of presentation was 73 years (range: 39–86 years). There was slightly higher male preponderance (Males 63.2% [*n* = 12] and females 36.8% [*n* = 7]). Most cases were reported from Europe (*n* = 9), followed by Asia (*n* = 5), North America (*n* = 3) and Australia (*n* = 1). Only five patients had a pre‐existing autoimmune disease, and one patient had pre‐existing von Willebrand disease. Most of the patients presented with spontaneous cutaneous hematomas followed by hemarthrosis with only three patients having major visceral hemorrhagic complications (melena, hemothorax, and hematuria). The most common location for cutaneous hematomas was the extremities followed by the trunk. Only one patient was found to have concurrent malignancy—pleomorphic dermal sarcoma, and only one patient developed concurrent immune‐mediated phenomenon—bullous pemphigoid. All AHA cases were reported after mRNA vaccines, BNT162b2 Pfizer‐BioNTech (*n* = 13) followed by mRNA‐1273 Moderna (*n* = 6) and none with adenoviral vaccination. The median time from the last administered vaccine dose to symptom onset was 14 days ‐11 patients presented after the second dose, seven patients after the first dose, and one after the booster dose. The overall mortality was 10.5% with two patients succumbing to complications—gall bladder rupture leading to sepsis/bleeding and acute respiratory distress, respectively. Majority of the patients were treated with either steroids alone or in a combination with recombinanat factor VIII (rFVIII). Escalation of therapy to add immunosuppressants (azathioprine, rituximab, cyclophosphamide) was required in 12 patients (63.15%). Only one patient was observed without treatment, and one patient just used rituximab alone as first line treatment.

The concept of vaccine‐associated hematological adverse events is not new, but with the advent of COVID‐19 pandemic, it has become a pressing issue to monitor such cases owing to the urgent need of counteracting the pandemic with successful mass scale anti‐SARS‐CoV2 vaccination campaign. As of May 21, 2022, 65.7% of the world population has received at least one dose of the COVID‐19 vaccine with a total of 11.76 billion doses administered [23]. Till May21, 2022, as per the pharmacovigilance database by the World Health Organization, over three million adverse events post‐COVID‐19 vaccine have been reported, most of which are nonserious. One hundred and fifty‐six cases of acquired hemophilia have been reported. [24].

Our review had similar demographics as found in the largest known AHA cohort called European Acquired Hemophilia registry where AHA was more commonly noted in older adults with median age 73.9 years with male predominance [25, 26]. AHA has been associated with autoimmune and dermatological conditions like systemic lupus erythematosus, rheumatoid arthritis, epidermolysis bullosa, and pemphigus [27]. A total of 26% of had pre‐existing diagnosis of immunological disorder—polymyalgia rheumatica, Sjogren syndrome, and Raynaud's phenomenon, but one patient developed concurrent bullous pemphigoid. The diagnosis of AHA can be challenging due to the rarity of this condition and complexity of laboratory workup. In all these studies, the authors reached a conclusion based on the temporal sequence of events and ruling out other causes of bleeding diathesis. Hence, we propose this schematic representation to aid in diagnosis [Figure 3].

Unlike SARS‐CoV2 adenoviral VITT for which mechanism has been well described, the pathophysiology behind mRNA‐ vaccine‐associated AHA is not well understood. Two possible mechanisms, antigen mimicry and stimulation of dormant T or B cells, have been proposed [Figure 2]. SARS‐CoV2 spike protein has 37% similarity with A2 domain at 540–570 amino acid position of factor VIII using NCBI blast sequence alignment tool and one overlapping epitome (FVIII 543–554) at the same sequence using silico antigenic peptide prediction [28]. Therefore, molecular mimicry was proposed in the immunopathogenesis of AHA following vaccination due to induction of antispike IgG antibodies that might act as FVIII inhibitor. Hirsiger et al. attempted to study this concept in three patients with AHA and found weak FVIII cross‐reactivity in antispike‐IgG‐enriched fraction [28]. Therefore, another hypothesis of activation of dormant T or B‐cells was proposed. MHC class II‐facilitated presentation of SARS‐CoV2 spike peptides to pre‐existing T cell clones specific to factor VIII can lead to their activation, resulting in production of autoantibodies. Polyclonal B cell can also be directly activated due to stimulation of broad Toll‐like receptors, with production of factor VIII‐specific antibodies of restricted isotypic heterogeneity. This is further supported by the fact that negative thymic selection of factor VIII‐specific CD4 T cells is incomplete, with high numbers of naïve and memory T cells, which can expand in response to peculiar immunogenic trigger resulting in AHA [29].

**FIGURE 2:**
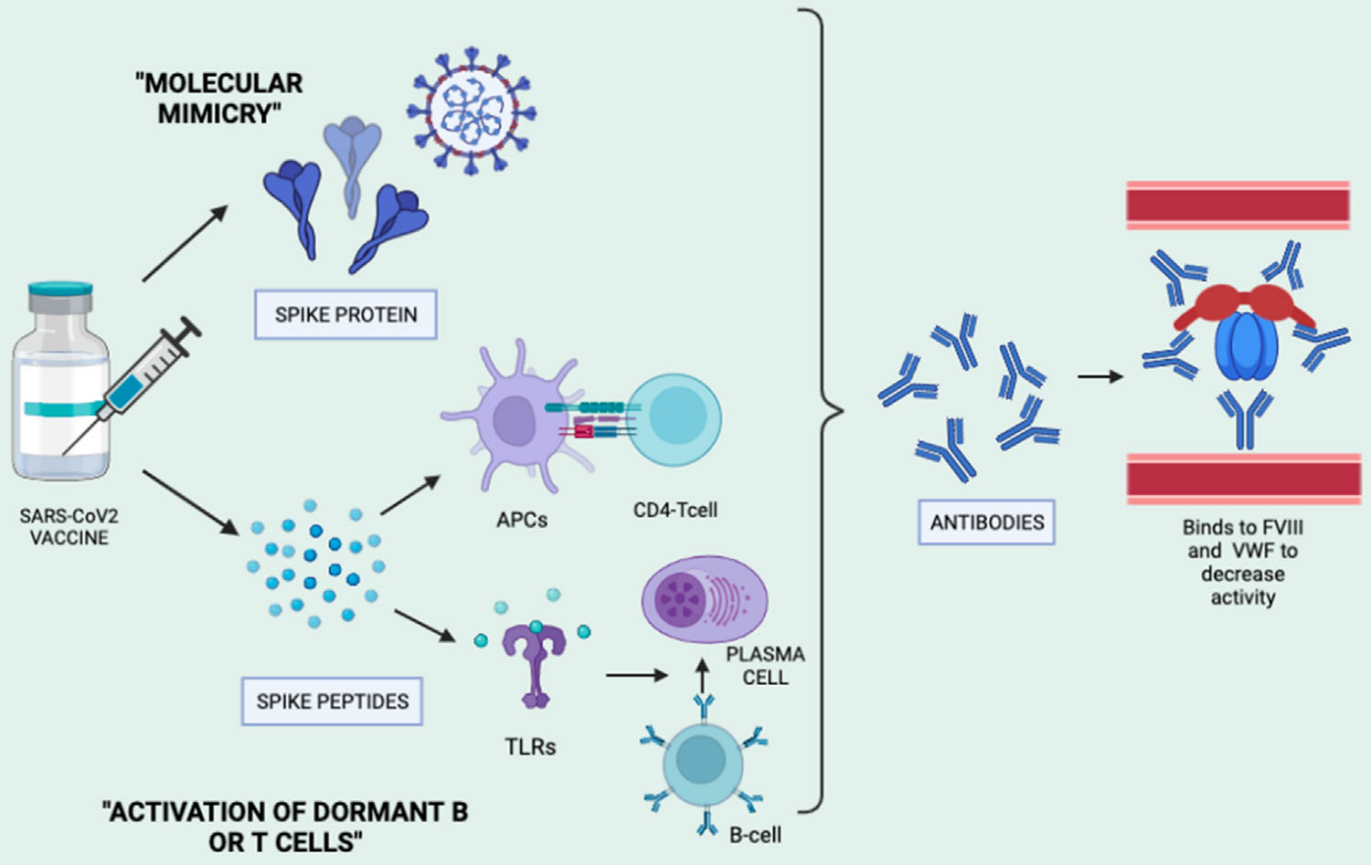
Representing possible pathophysiological cause of vaccine‐induced acquired hemophilia A (AHA). Image created in biorender.com

**FIGURE 3:**
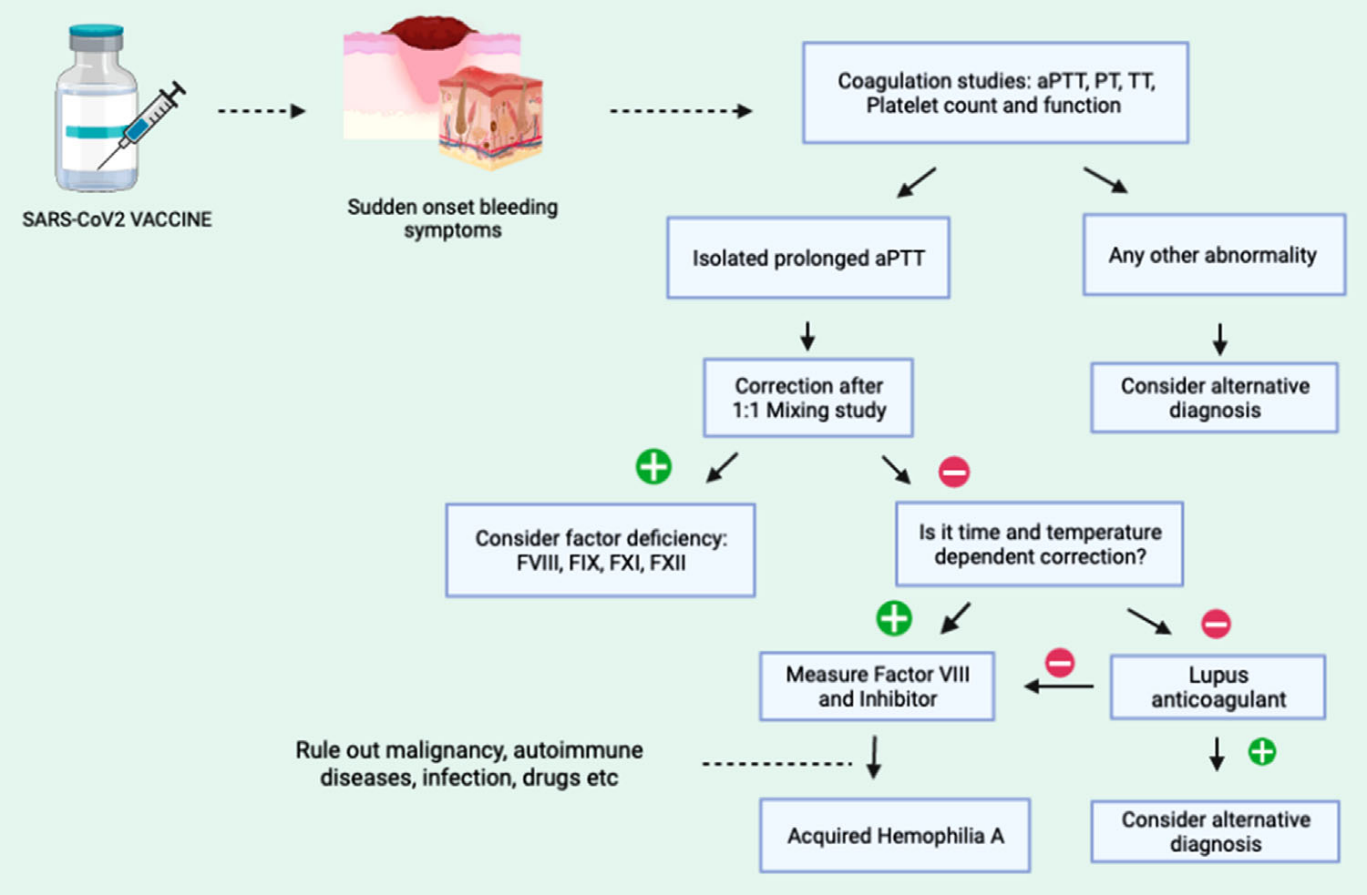
Representing the laboratory workup for diagnosis of vaccine‐induced acquired hemophilia A (AHA). Image created in biorender.com

Although another study done by Algiman et al. showed that anti‐VIII IgG were present in healthy individuals without evidence of disease, these could merely represent natural IgG autoantibodies or antibodies against some epitopes with unknown allotypic polymorphism of factor VIII. In the setting of polyclonal activation, some AHA predisposed people can still expand these self‐reactive clones or somatically mutated antigen driven B cell clones producing pathogenic IgG autoantibodies causing clinical disease [30]. In our study, bleeding occurred within 1–3 weeks of receiving vaccines, with bleeding being more severe in those who completed the two doses, which raises the suspicion of additive effect of antigen exposure leading to the outcome. Further understanding is required into the fact that no cases have been reported with adenoviral vaccination, whether that is a coincidence, or a reality is unknown.

There are two major goals of therapy: control of bleeding and neutralizing the inhibitor. For mild bleeding in nonsignificant organ or area, observation is preferred. For significant moderate to severe bleeding, either APCC or rFVIII is used. As per 2020 international AHA guidelines, early initiation of immunosuppressive therapy to eliminate the inhibitor has improved outcomes [31]. As per GTH study on AHA in 2010 (German Society of Thrombosis and Hemostasis research group), corticosteroids should be started for 3 weeks or till the achievement of partial remission. They define partial remission as factor VIII activity being >50% without needing any blood products and in the absence of any active bleeding. If partial remission cannot be achieved, initiation of cyclophosphamide around 4–6th week followed by rituximab from 7–10th week is recommended. However, the use of immunosuppressive therapy has to be done cautiously if World Health Organization performance status is poor on presentation as Acquired Hemophilia Working Group of the German, Austrian and Swiss Thrombosis and Hemostasis Society study has reported that the risk of immune system suppression‐related mortality especially due to infection is higher than the risk of life‐threatening bleeding due to AHA [32]. The use of immunosuppressants result in 60%–90% remission rates, but mortality rates can be as high as 28%–42% due to direct complications of bleeding or infection due to immunosuppressants [32]. In our review, two patients died due to gall bladder rupture and acute respiratory distress syndrome respectively. The other patients had favorable short term prognosis in terms of safe discharge from hospital and no bleeding‐related life threatening complications. Most patients responded to the treatment, but long‐term outcome for these patients is lacking and needs more studies in future to draw succinct conclusions.

Although there is a definite temporal association in the absence of any other inciting factors, causation cannot be proven. This was further corroborated by a study done by Cittone et al. who reported no statistically significant increase in AHA incidence in Switzerland during anti‐ SARS‐CoV‐2 vaccination campaign [11]. More evidence in the form of prospective studies is needed to prove the causation. The potential areas that need further exploration include whether these patients should receive the next dose of vaccine, and if yes, whether the same vaccine can be administered. It would be helpful if we will be able to identify and predict biomarkers as well. In the meantime, a shared decision making with the patients can be done. If a decision is taken to re‐vaccinate with second dose or boosters, monitoring of the coagulation studies near the vaccination administration period should be undertaken and patients should be educated regarding the warning signs to present to the hospital in the event of occurence of any bleeding episodes. This study has several limitations including short time of disease emergence and postmarketing surveillance. There is always a concern of under‐reporting, publication bias, and missing articles with any systematic review of case reports. We also have to acknowledge that some cases might have not been detected or might have self‐resolved before a diagnosis could have been established.

In conclusion, the number of cases reported AHA since the beginning of COVID‐19 vaccination is not significant enough to have definitive causal association between the two but allows us to direct more attention to epidemiological data on suspected vaccine‐related adverse events. Nonetheless, COVID‐19 vaccination benefits far exceed the risk of potential immune‐mediated hematological side effects as far as individual and public health is concerned, but AHA should always be kept in differential while evaluating a patient with bleeding diathesis post‐COVID 19 vaccination.

## AUTHOR CONTRIBUTIONS

*Conceptualization*: FA and VR. *Methodology*: FA and PS. *Formal analysis*: FA and PM. *Writing—original draft preparation*: FA. *Writing—review and editing*: PS, FA, and PM. *Supervision*: VR. *Read and approved the submitted and final versions of the manuscript*: FNUA, PS, PM, and FVR.

## CONFLICT OF INTEREST

The authors declare they have no conflicts of interest.

## FUNDING INFORMATION

The authors received no specific funding for this work.

## Data Availability

The authors declare that all data supporting the findings of this study are available within the article or in the article mentioned in the references.
